# Trehalose Interferes with the Photosynthetic Electron Transfer Chain of *Cereibacter (Rhodobacter) sphaeroides* Permeating the Bacterial Chromatophore Membrane

**DOI:** 10.3390/ijms252413420

**Published:** 2024-12-14

**Authors:** Giovanni Venturoli, Mahir D. Mamedov, Liya A. Vitukhnovskaya, Alexey Y. Semenov, Francesco Francia

**Affiliations:** 1 Department of Pharmacy and Biotechnology, University of Bologna, Via Irnerio n.42, 40126 Bologna, Italy; giovanni.venturoli@unibo.it; 2 Consorzio Nazionale Interuniversitario per le Scienze Fisiche della Materia (CNISM), c/o Dipartimento di Fisica e Astronomia (DIFA), Università di Bologna, Via Irnerio 46, 40126 Bologna, Italy; 3 A.N. Belozersky Institute of Physical-Chemical Biology, Moscow State University, Moscow 119991, Russia; mahirmamedov@yandex.ru (M.D.M.); vitlia@yahoo.com (L.A.V.); aysemenov51@gmail.com (A.Y.S.)

**Keywords:** trehalose, chromatophores, *Cereibacter sphaeroides*, electrometrical technique, time-resolved spectrophotometry, reaction center, cytochrome *bc*_1_

## Abstract

Disaccharide trehalose has been proven in many cases to be particularly effective in preserving the functional and structural integrity of biological macromolecules. In this work, we studied its effect on the electron transfer reactions that occur in the chromatophores of the photosynthetic bacterium *Cereibacter sphaeroides*. In the presence of a high concentration of trehalose, following the activation of the photochemistry by flashes of light, a slowdown of the electrogenic reactions related to the activity of the photosynthetic reaction center and cytochtome (cyt) *bc*_1_ complexes is observable. The kinetics of the third phase of the electrochromic carotenoid shift, due to electrogenic events linked to the reduction in cyt b_H_ heme via the low-potential branch of the cyt *bc*_1_ complex and its oxidation by quinone molecule on the Q_i_ site, is about four times slower in the presence of trehalose. In parallel, the reduction in oxidized cyt (*c*_1_ + *c*_2_) and high-potential cyt *b*_H_ are strongly slowed down, suggesting that the disaccharide interferes with the electron transfer reactions of the high-potential branch of the *bc*_1_ complex. A slowing effect of trehalose on the kinetics of the electrogenic protonation of the secondary quinone acceptor Q_B_ in the reaction center complex, measured by direct electrometrical methods, was also found, but was much less pronounced. The direct detection of carbohydrate content indicates that trehalose, at high concentrations, permeates the membrane of chromatophores. The possible mechanisms underlying the observed effect of trehalose on the electron/proton transfer process are discussed in terms of trehalose’s propensity to form strong hydrogen bonds with its surroundings.

## 1. Introduction

Among sugars, disaccharide trehalose (α-D-glucopyranosyl-α-D-glucopyranoside) has been found to be most effective in enhancing the stability of proteins in vitro both in solutions [1,2,3,4,5,6,7] and in the dry state [8,9,10,11,12,13,14], counteracting the loss of native conformation and function caused by harsh environmental conditions such as high/low temperatures and/or desiccation. In nature, trehalose is synthesized in large amounts by several organisms (plants, invertebrates, and microorganisms) that are able to survive extreme dehydration and high temperatures by entering a state called anhydrobiosis, in which their metabolic activity is reversibly suspended [15]. In the phenomenon of anhydrobiosis, trehalose has been recognized to play a central role by exerting an extraordinary bioprotective action on membranes and protein complexes [3,8]. Despite an extensive body of experimental and in silico studies, the molecular basis of the peculiar effectiveness of trehalose in stabilizing biomolecules both in vivo and in vitro is still far from being understood [13]. Different molecular models (often complementary and not mutually exclusive) have been proposed in which the prevailing factor responsible for stabilization varies from direct hydrogen bonding between trehalose and exposed protein groups (water replacement hypothesis) [16,17,18,19] to the retainment and confinement by trehalose of the protein hydration shell (water entrapment hypothesis) [20,21,22,23,24,25]. Whatever the detailed molecular mechanism, the peculiar bioprotective properties of trehalose are likely to be related to its unique chemical–physical properties, i.e., (i) its unusually high glass transition temperature favoring room temperature vitrification at low humidity [26]; (ii) a stronger interaction strength with water molecules resulting in the strong perturbation of the tetrahedral intermolecular H-bond network of pure water and in a more extended hydration than other disaccharides [27,28,29,30,31]; (iii) a more pronounced slowing down of water dynamics as compared to other saccharides [22,23,32].

The distinctive properties of trehalose summarized above have been exploited by our group in a series of studies focused on the effects of trehalose on membrane proteins purified from photosynthetic systems both under fully hydrated (buffer solution) and partially dehydrated (amorphous solid matrices) conditions.

The use of amorphous trehalose matrices incorporating reaction centers from purple bacteria [33,34,35,36] or photosystem I from cyanobacteria [37] has allowed to progressively retard the conformational dynamics of the embedded protein complexes upon decreasing the hydration of the system. This resulted in the progressive inhibition of specific electron transfer reactions, mimicking, at room temperature, the behavior of the systems at cryogenic temperature, and providing information on the role of internal conformational dynamics in electron transfer processes [34,37].

In a different series of studies performed in fully hydrated systems, i.e., in room temperature buffer solutions of photosystem (PS) I and II, we examined the effects of trehalose on specific partial reactions catalyzed by photosystems and investigated the ability of trehalose to stabilize protein complexes by preserving their function for long times at room temperature.

We found that trehalose stimulates the steady-state rate of oxygen evolution in PS II complexes [38]. The decay kinetics of flash-induced fluorescence demonstrated that trehalose did not affect the oxidation rate of the primary electron acceptor Q_A_^−^, but rather increased the relative fractions of PS II reaction centers capable of Q_A_^−^ oxidation [38]. It was proposed that trehalose, affecting the extent of hydration, favors a protein conformation which optimizes photoinduced electron transfer. A complimentary study was performed in Mn-depleted PS II preparations (apo-WOC-PSII), which are characterized by a significantly increased rate of oxygen photoconsumption (OPC) [39,40], most likely associated with the formation of organic hydroperoxides on the electron donor side of PS II [41]. In apo-WOC-PSII preparations, the addition of 1 M trehalose resulted in a more than two-fold increase in the OPC [42]. The drastic (30–70%) inhibition of OPC upon the addition of either an electron acceptor or electron donor indicated that the trehalose-induced OPC activation occurs on both the donor and acceptor sides of PS II and suggested that it is related to trehalose-induced conformational changes leading to an increased electron transfer rate within PS II and facilitating the access of molecular oxygen to components of the electron transport chain on the acceptor and donor sides of the complex [42]. In a subsequent study [43], by measuring light-induced changes in chlorophyll fluorescence yield, we found that 1 M trehalose enhanced the Mn^2+^-dependent suppression of the photoinhibition of apo-WOC-PSII. It was concluded that trehalose specifically increased the capability of manganese (both exogenous and endogenous) to donate electrons to apo-WOC-PSII reaction centers, thus amplifying the protective properties of Mn against the photoinhibition of apo-WOC-PSII, probably due to trehalose-induced conformational changes affecting the donor side of PS II. More recently [44], the trehalose-induced stimulation of the steady-state oxygen evolution rate was further investigated in spinach PS II core complexes reconstituted into lipid vesicles (proteoliposomes) in which the light-induced vectorial transfer of charges generates a transmembrane electric potential difference. By using a direct electrometrical technique, it was found that trehalose had no effect on the kinetics of electron transfer from Mn to redox-active-tyrosyl radical, Y_Z_^•^ (S_1_ → S_2_ transition), while it accelerated the kinetics of electrogenic proton transport during S_2_ → S_3_ and S_4_ → S_0_ transitions of the WOC. Taken together, the results mentioned above suggest that, in the presence of high trehalose concentrations, the catalytic site at the donor side of PS II assumes a conformation which facilitates the release of its products (O_2_ and protons).

As to the bioprotective effects exerted by trehalose on photosynthetic reaction center (RC) complexes, they have been recently tested by examining steady-state voltage generation induced by continuous illumination in a system consisting of purified PS II [45] or PS I complexes [46] immobilized onto a nitrocellulose membrane filter sandwiched between two semiconductor indium tin oxide electrodes. In the simple PS II-based bio-hybrid solar converter, trehalose was found essential for maintaining the ability to generate a stable voltage on the time scale of hours [45]. Remarkably, the PS I complex immobilized within the membrane porous filter retained its functionality for at least 6 months at room temperature in the presence of highly concentrated trehalose [46]. The ability to generate a stable photoelectrical response under continuous illumination was also demonstrated for chromatophores from the photosynthetic bacterium *Cereibacter (Cba) sphaeroides* (previously *Rhodobacter sphaeroides*) immobilized onto a membrane filter [47].

Based on the above summarized results, it appears that trehalose, besides its beneficial stabilizing effects on photosynthetic protein complexes, can significantly affect electron and/or proton transfer processes catalyzed by photosystems, not only in amorphous matrices at low humidity, but even in room temperature solutions. A detailed knowledge of these effects is highly desirable when considering the promising use of trehalose for the long-time functional preservation of photosynthetic complexes in bio-hybrid energy-converting devices (see e.g., [45,46,47,48,49]).

In the present work, we have focused on the effect of highly concentrated trehalose on the partial reactions of the light-induced cyclic electron transfer chain of the photosynthetic bacterium *Cba. sphaeroides,* as studied in chromatophores, i.e., in intracytoplasmic vesicles, which can be easily isolated from several species of photosynthetic purple bacteria [50]. In the study of photosynthetic processes, chromatophores represent an ideal model system which offers distinctive advantages, among which are (a) the chromatophore membrane accommodates all the integral protein complexes which constitute the photosynthetic apparatus in their native phospholipidic environment; (b) most of the light-induced electron transfer processes can be monitored directly by visible absorption spectroscopy without any *a priori* limitations in time resolution because the cyclic electron transfer chain can be elicited by short flashes of light; (c) the generation of the transmembrane electrical potential difference (Δψ) arising from the vectorial character of several electron transfer reactions, and/or proton movement, leading to charge displacement across the membrane dielectric, can be conveniently followed in real time by means of an endogenous spectral probe acting as an intramembrane voltmeter [51,52]; in fact, the visible spectrum of carotenoids bound to the antenna complexes [53] undergoes an electrochromic redshift, which results at the appropriate wavelengths in absorbance change signals (carotenoid shift) responding linearly to the intramembrane electric field [51]; (d) electrogenic processes within the chromatophore membrane can be alternatively monitored by using a direct electrometric approach based on the absorption of chromatophores to a phospholipid-impregnated collodion film interposed between two electrolyte compartments hosting Ag/AgCl macroelectrodes (see Section 4.2 and [54,55,56]). A time-resolved electrometrical technique is a direct approach to obtain information on kinetics and dielectrically weighted distances of electrogenic charge transfer reactions [57,58].

**Figure 1:**
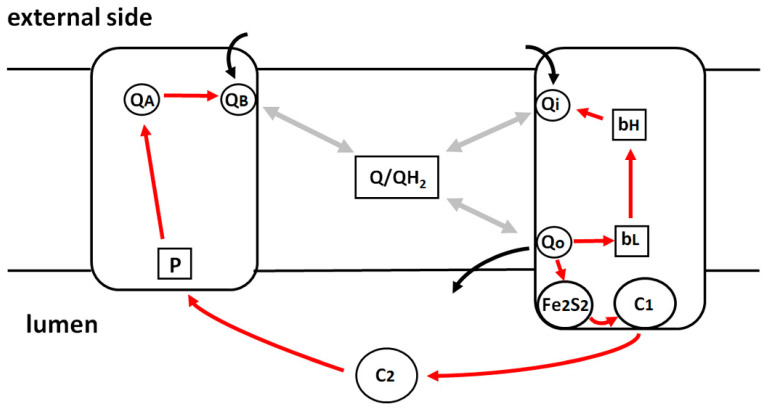
Simplified cartoon representation of the cyclic electron transfer chain of the photosynthetic bacterium *Cereibacter sphaeroides.* Q_o_ and Q_i_, quinol oxidation and quinone reduction sites of the cyt *bc*_1_ complex, respectively; Fe_2_S_2_, iron sulfur cluster of the ISP; *c*_1_ and *c*_2_, cytochrome *c*_1_ and *c*_2_; *b*_L_ and *b*_H_, cytochromes *b*_L_ and *b*_H_; P, bacteriochlorophyll special dimer of the RC; Q_A_ and Q_B_, primary and secondary quinone electron acceptor of the RC, respectively. Red lines indicate electron transfer, black lines represent proton uptake or release, and thick gray lines represent the equilibria between oxidized (Q) and/or reduced (QH_2_) quinone molecules from the pool with interaction sites of the RC and the cyt *bc*_1_ complexes.

The main components involved in the light-induced electron transfer chain of *Cba. sphaeroides* are represented schematically in Figure 1. Cyclic electron transfer is catalyzed by two integral membrane proteins, RC and cyt *bc*_1_ complex [59]. Redox interaction between the two complexes is mediated by two diffusible electron carriers: the water soluble cyt *c*_2_ trapped inside the chromatophore vesicle (lumen) and a pool of ubiquinone-10 (Q) molecules dissolved in the hydrophobic moiety of the phospholipid bilayer and present in a large stoichiometric excess over the cyt *bc*_1_ complex. Upon the absorption of a photon, the primary electron donor P, a pair of bacteriochlorophyll molecules bound on the RC side facing the chromatophore lumen, acts as the primary electron donor by delivering an electron to the primary quinone acceptor Q_A_ bound at an RC site close to the external side of the membrane. This primary charge separation, accomplished in about 200 ps, is followed by electron transfer from Q_A_^−^ to the secondary quinone acceptor Q_B_ taking place in tens of microseconds. The photooxidized P^+^ is rapidly reduced by cyt *c*_2_ so that a second photochemical turnover of the RC can take place, leading to double reduction in Q_B_, which protonates to ubiquinol by proton uptake from the external aqueous phase. The QH_2_ thus produced at the Q_B_ site is released by the RC into the phospholipid bilayer and equilibrates with the Qpool. QH_2_ from the pool or released by the Q_B_ site is oxidized at the Q_o_ site of the cyt *bc*_1_ complex, located on the lumen side. According to the Q-cycle mechanism [60,61], the first electron resulting from QH_2_ oxidation at the Q_o_ site enters the high-potential redox chain of the complex, reducing in sequence the Fe_2_S_2_ center (located in the hydrophilic domain of the cyt *bc*_1_ Iron Sulfur Protein (ISP)) and cyt *c*_1_, which, in turn, donates the electron to cyt *c*_2_ oxidized by the P^+^ of the RC, thus closing the cyclic electron transfer chain. The soluble electron carrier cyt *c*_2_ shuttles between the RC and the cyt *bc*_1_ complex by diffusing at the membrane–lumen interface. The second electron resulting from semiquinone oxidation is delivered sequentially to heme *b*_L_ and heme *b*_H_, from which it is transferred to ubiquinone or semiquinone bound at the Q_i_ site located on the opposite (external) side of the membrane. QH_2_ oxidation at the Q_o_ site and Q reduction at the Q_i_ site are associated with proton release and uptake, respectively.

By monitoring, in parallel, the generation of Δψ and the redox state of cytochromes *b* and *c* following the single turnover flash excitation of the cyclic electron transfer chain, we found that the addition of trehalose to the chromatophore suspension significantly affects the kinetics of electron and proton transport steps catalyzed by the cyt *bc*_1_ and RC complexes, respectively. The results obtained imply that trehalose permeates the chromatophore membrane.

## 2. Results

### 2.1. Effects of Trehalose on the Reduction and Oxidation Sites of the RC Complex

In this section, the effect of trehalose on the light-induced protonation of the doubly reduced quinone bound at the Q_B_ site of the RC and on the oxidation of cytochrome *c*_2_ by the photooxidized primary electron donor P^+^ of the RC are examined by means of the direct electrometric approach (see Section 1 and Section 4.2).

#### 2.1.1. Effects of Trehalose on the Protonation of the Doubly Reduced Quinone Bound at the Q_B_ Site

**Figure 2:**
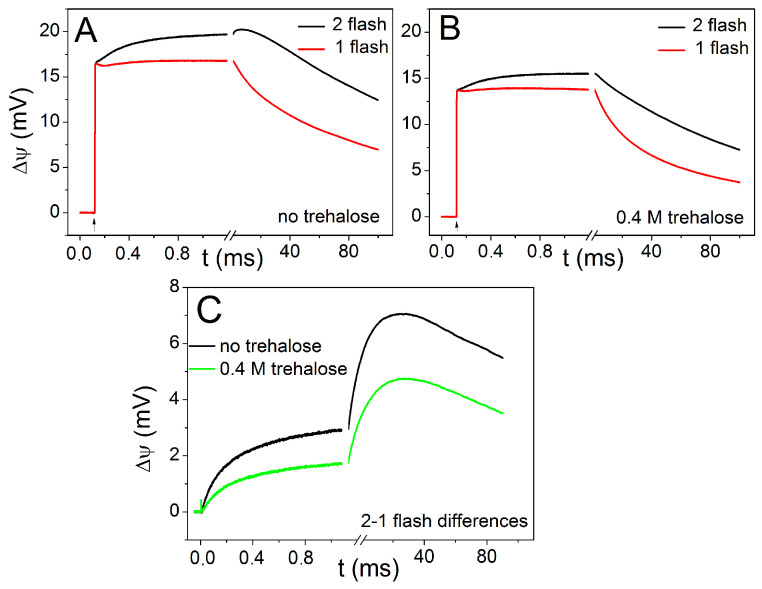
Generation of electric potential difference in chromatopohores attached to the phospholipid collodion membrane. (**A**) Photoresponses induced by the first (red trace) and second (black trace) laser flash in the presence of 2 mM ferrocyanide, 50 μM TMPD, and 20 mg/mL CoQ_10_ in the phospholipid membrane. Incubation medium: 25 mM HEPES-NaOH (pH 7.5). (**B**) The corresponding photoelectric signals in the presence of 0.8 M trehalose: first flash (red trace), second flash (black trace). (**C**) Difference between photoresponses induced by the first and the second flashes in the absence (black trace) and in the presence (green trace) of 0.8 M trehalose. The time lag between the first and the second flashes is 2 s. The experiments were repeated 4 times for different samples of chromatophores. The figure shows the result of typical experiment. A minimum dark time of 120 s was allowed between series of two consecutive flashes.

Figure 2A shows the laser-flash-induced generation of Δψ following the first and the second flash in chromatophores attached to the phospholipid collodion membrane in the presence of added CoQ_10_. The photoelectric signal was measured under oxidizing conditions (in the presence of N,N,N′,N′-tetramethyl-p-phenylenediamine (TMPD) and potassium ferrocyanide) when the Qpool was fully oxidized before the series of flashes (E_h_ = 300 mV), while cyt *c*_2_ was partially reduced. The first flash induces the kinetically unresolved transmembrane charge separation between the bacteriochlorophyll special pair P and the secondary ubiquinone acceptor Q_B_ via bacteriopheophytin and the primary quinone Q_A_. The small Δψ rise in the sub-millisecond time scale reflects electrogenic electron transfer between the partially reduced cyt *c*_2_ and P^+^. The photoelectric response upon the second flash contains an additional component of Δψ rise in the sub-millisecond time scale due to the electrogenic protonation of the double-reduced Q_B_^2−^, which is followed by the electrogenic reactions in the cyt *bc*_1_ complex in the slower time range (tens of ms). Figure 2B shows a similar experiment in the presence of 0.8 M trehalose. The comparison of the data presented in Figure 2A,B demonstrates the trehalose-induced decrease in the very fast phase of Δψ generation and the acceleration of decay induced by both the first and second flashes. Figure 2C shows the difference between the second and the first flash in the presence and in the absence of trehalose. The kinetic analysis of the traces presented in Figure 2C reveals two main exponential components with characteristic times τ_1_ 0.18 ± 0.03 ms and τ_2_ 12 ± 2.6 ms and relative amplitudes accounting for 27% and 73% of the signal, respectively. These components were previously ascribed to the electrogenic protonation of Q_B_ and electrogenic reactions of the cyt *bc*_1_ complex, respectively [62,63]. In the presence of 0.8 M trehalose, the decrease in and slowing of both components by ~25% can be observed.

#### 2.1.2. Electron Transfer from Cytochrome c_2_ to the Photooxidized Primary Electron Donor P^+^ of the RC

**Figure 3:**
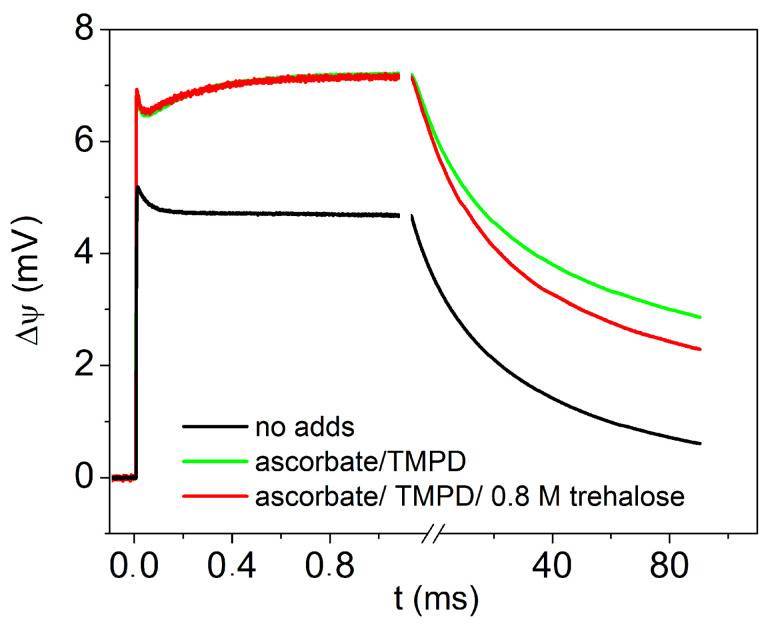
Generation of electric potential difference by chromatophores attached to the phospholipid collodion membrane under single laser flash excitation. Black trace—photoelectric response of chromatophores without additions; green trace—in the presence of 2.5 mM ascorbate and 150 μM TMPD; red trace—in the presence of 2.5 mM ascorbate, 150 μM TMPD, and 0.8 M trehalose after 2 h of incubation. Incubation medium as in Figure 2.

Figure 3 shows the electrometric experiment in the absence of CoQ_10_ in the phosholipid membrane. Measurements have been performed in the absence of any additions and following the addition of sodium ascorbate (Asc) in combination with the redox mediator TMPD. Under the latter conditions, the internal electron donor cyt *c*_2_ is almost fully reduced before the laser flash. The black trace shows the photoelectric response of chromatophores in the absence of additions, and green trace in the presence of Asc and TMPD. As can be seen, the addition of Asc and TMPD leads to an increase in the very fast unresolved phase of Δψ generation due to charge separation between P^+^ and the primary quinone acceptor Q_A_^−^, and the appearance of an additional rise phase of Δψ in the sub-millisecond time scale due to the reduction in P^+^ by cyt *c*_2_ [63]. Measurements after incubation for two hours in the presence of 0.8 M trehalose result in a minor increase in the amplitude of the Δψ component due to the cyt *c*_2_ → P^+^ electron transfer (red trace). The kinetic analysis shows that the sub-millisecond rise phase ascribed to the oxidation of cyt *c*_2_ is monoexponential and exhibits a characteristic time τ 0.33 ± 0.03 and 0.37 ± 0.03 ms in the absence and in the presence of trehalose, respectively.

### 2.2. Effects of Trehalose on the Electron Transfer Processes Catalyzed by the cyt bc_1_ Complex

This section deals with the effects of trehalose on the individual electron transfer steps involving the redox centers of the cyt *bc*_1_. These effects have been studied by using time-resolved spectrophotometry to follow the kinetics of redox changes induced by single turnover photoexcitation in selected redox centers of the cyt *bc*_1_ complex, or to monitor the build-up of Δψ associated with specific electrogenic partial reactions through the electrochromic shift of endogenous carotenoids (see Section 1 and Section 4.3).

#### 2.2.1. Effect of Trehalose on the Flash-Induced Electrochromic Carotenoid Signal

**Figure 4:**
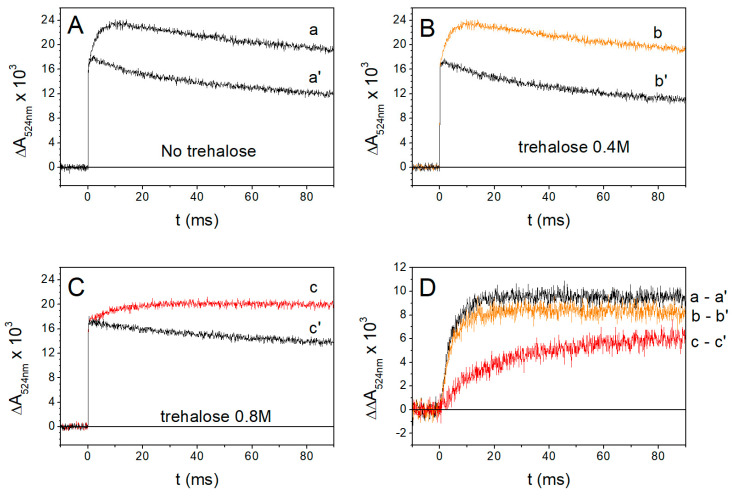
Panels (**A**–**C**). Kinetics of the carotenoid shift induced by a single turnover flash, recorded at 524 nm, in chromatophores under reducing conditions in the absence (traces a, b, c) or presence of 10 μM antimycin (traces a’, b’, c’). See Section 4.3 of Material and Methods for the detailed composition of the assay medium. Panel (**A**): control sample, without trehalose; panel (**B**): in the presence of 0.4 M trehalose; panel (**C**): in the presence of 0.8M trehalose. Panel (**D**) shows the kinetics of the antimycin-sensitive phase III of the CS, obtained by subtracting the traces in the presence of antimycin (a’, b’, c’) from the corresponding traces recorded in the absence of the inhibitor (a, b, c). Traces are the average of four individual signals. A minimum dark time of 60 s was allowed between repetitive photoexcitation during averaging.

Following the generation of Δψ induced by RC photoexcitation via an actinic flash of light, carotenoids contained in light-harvesting complex II undergo an electochromic redshift of their absorption spectrum. As the amplitude of the carotenoid band shift is proportional to Δψ, monitoring its time evolution at appropriate wavelengths allows us to study the kinetics of the electrogenic events induced in chromatophores by the activation of photochemistry [51,52]. Figure 4 shows the kinetics of the carotenoid shift signal (CS), recorded at 524 nm, induced by a single turnover flash in chromatophore suspensions under reducing conditions in the absence (panel A) and in the presence of trehalose at 0.4 M (panel B) and 0.8 M concentration (panel C).

The rise of the CS consists of three kinetic phases [52,64]. The first two phases (I and II), insensitive to inhibitors of the *bc*_1_ complex, originate from charge displacement localized in the RC complex, associated with electron transfer from cyt *c*_2_ to the final ubiquinone acceptor Q_B_ (see Figure 1): phase I is ascribed to the vectorial electron transfer from P to Q_A_ [52], while phase II includes contributions from electron transfer from cyt *c*_2_ to the photooxidized P^+^ [52] and from the protonation of the secondary quinone acceptor Q_B_ [65]. These two phases, completed in a few tens of microseconds, are visible as a not resolved instantaneous increase in absorbance in the CS traces of Figure 4, panels A, B, C. In the absence of inhibitors of the cyt *bc*_1_ complex (traces a, b, c), the fast CS onset (phase I plus phase II) is followed by the slower phase III rise, which occurs in the millisecond time scale, and is due to charge displacements taking place within the cyt *bc*_1_ complex [64]. In the presence of the cyt *bc*_1_ inhibitor antimycin, which blocks the vectorial electron transfer from *bc*_1_ heme *b*_H_ of the cyt *b* chain of the complex to the quinone bound at the Q_i_ site (see Figure 1); the amplitude of phase III is drastically reduced [66], as shown by traces a’, b’, c’. The onset kinetics of the antimycin-sensitive phase III, obtained by subtracting the CS signal in the presence of antimycin from the signal recorded in the absence of the inhibitor, are shown in Figure 4 panel D for chromatophore in the absence (trace a-a’) and in the presence of 0.4M (trace b-b’) and 0.8 M trehalose (trace c-c’). In the presence of 0.8 M trehalose, phase III kinetics is significantly slowed down compared to the control (Figure 4D, red and black traces, respectively), while only a very small decrease in the amplitude of the phase is observable in the presence of 0.4 M trehalose (Figure 4D, orange trace). From the traces of Figure 4D, it can be estimated that the half-time of phase III rise, approximately equal to 2.8 ms for the control and in the presence of 0.4M trehalose increases to 12.6 ms in the presence of 0.8M trehalose. This effect points to a significant slowing of electron transfer along the cyt *b* chain upon the oxidation of QH_2_ at the Q_o_ site of the *bc*_1_ complex.

#### 2.2.2. Reduction Kinetics of cyt b_H_ Heme in the Presence of Trehalose

In view of the slowing of CS phase III described above, with the aim of identifying which specific partial reaction occurring within the cyt *bc*_1_ complex is affected by trehalose at high concentration, we studied the effect of trehalose on the kinetics of the reduction in cyt *b*_H_ induced by RC photoexcitation in the presence of specific inhibitors of the cyt *bc*_1_ complex. In the presence of antimycin, a powerful inhibitor of the Q_i_ site, the electron can no longer be transferred from the heme *b*_H_ to the oxidized quinone or semiquinone bound at the Q_i_ site, and the kinetics of heme *b*_H_ reduction, reflecting the oxidation of QH_2_ at the Q_o_ site, can be studied (see Section 1, Figure 1).

**Figure 5:**
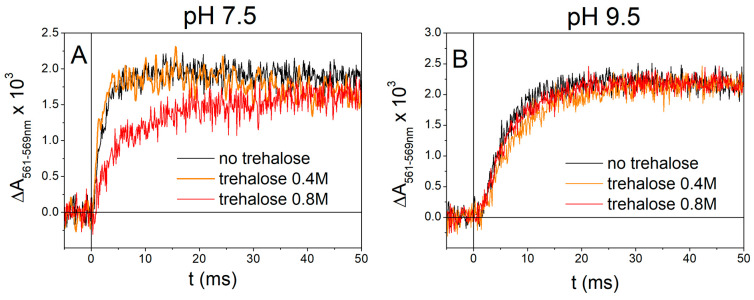
Reduction kinetics of heme *b*_H_ in the presence of the inhibitors of the Q_i_ (panel **A**) or of the Qo (panel **B**) site. Flash-induced redox changes in cyt b_H_ have been measured at 561–569 nm in chromatophores resuspended in 25 mM Na-Hepes buffer, pH 7.5, in the presence of 10 μM antimycin (panel **A**) or in 50 mM Na-CHES buffer, pH 9.5, plus 2 μM myxothiazol (panel **B**). Black traces have been recorded in the absence of trehalose, and orange and red traces in the presence of 0.4 M and 0.8 M trehalose, respectively. See text and Section 4.3 of Materials and Methods for details. Traces are the average of 32 individual signals for each of the two wavelengths. A minimum dark time of 60 s was allowed between repetitive photoexcitation during averaging.

Figure 5A shows the kinetics of heme *b*_H_ reduction resulting from quinol oxidation at the Q_o_ site in chromatophores with antimycin-inhibited cyt *bc*_1_ complexes. Very similar kinetics are observed in the absence of trehalose or in the presence of 0.4 M trehalose (with estimated half-times of about 1.6 ms and 1.2 ms, respectively), indicating that 0.4 M does not significantly affect catalysis on the Q_o_ site. At variance, the presence of trehalose at 0.8 M concentration causes a marked retardation of cyt *b*_H_ reduction kinetics, characterized by an increased half-time of about 4.8 ms. These results are in line with what was observed on the kinetics of the antimycin-sensitive phase III of the CS (Figure 4D), i.e., an essentially negligible effect of 0.4 M trehalose, and a considerable slowing in the presence of 0.8 M trehalose. Notably, at the higher trehalose concentration, the extents of the slowdown of phase III and cyt *b*_H_ kinetics were comparable (by a factor of about 4.5 and 4.3, respectively). The direct observation of a slowed reduction in heme *b*_H_ in the presence of antimycin indicates clearly that the delivery of the second electron resulting from QH_2_ oxidation at the Q_o_ site to the cyt *b* chain of the complex is substantially affected by 0.8 M trehalose.

Since the antimycin-sensitive phase III of the CS reflects the electrogenic protonation of the quinone in the Q_i_-site of the complex [67], we cannot exclude that either electron transfer between *b*_L_ and *b*_H_ hemes or protonation itself is affected by trehalose at high concentrations, implying that trehalose also perturbs the catalytic activity of the Q_i_ site. This occurrence is, in principle, plausible when considering that Q_i_ is located near the chromatophore side exposed to the external solvent (see Figure 1). Externally added trehalose could, in principle, interfere with the proton uptake associated with the reduction in oxidized quinone or semiquinone by heme *b*_H_ at the Q_i_ site [68]. In order to test the possibility that trehalose interferes with the catalytic activity of the Q_i_ site, we examined the kinetics of cyt *b*_H_ reduction through the Q_i_ site in the presence of myxothiazol. In the presence of this potent inhibitor of the Q_o_ site, in fact, at basic pH and when the quinone pool is totally oxidized, the reduction in heme *b*_H_ due to the oxidation of QH_2_ released by the Q_B_ site of the reaction center at the Q_i_ site can be observed [69]. As shown in Figure 5B, the reduction kinetics of heme *b*_H_ through the Q_i_ site is not affected significantly by the presence of trehalose. Half-times of approximately 4.9 ms, 6.2 ms, and 5.1 ms in the absence of trehalose, in the presence of 0.4 M trehalose, and in the presence of 0.8 M trehalose, respectively, can be estimated. Although the reduction reaction of heme *b*_H_ through the Q_i_ site involves an inverse mechanism in that the reduced quinone generated by the photoactivity of the RC must deprotonate and donate the electron to the heme *b*_H_, the substantial absence of any effect exerted by trehalose suggests that the catalysis carried out by cyt *bc*_1_ at the Qo site, and not that carried out at the Q_i_ site, is mainly sensitive to the presence of trehalose at a high concentration.

#### 2.2.3. Dissection of the Third Phase of the Carotenoid Shift into the Antimycin-Sensitive and Myxothiazol-Sensitive Phase

Myxothiazol is an inhibitor of the Q_o_ site of the *bc*_1_ complex, which blocks the reduction in both *b*_L_ and *b*_H_ hemes as well as in the Fe_2_S_2_ center [70,71]. It has been shown that myxothiazol inhibits a portion of phase III of the carotenoid shift, which is insensitive to antimycin [66]. This additional component of phase III, antimycin-insensitive but myxothiazol-sensitive, is attributed to the vectorial electron transfer from heme *b*_L_ to heme *b*_H_ of the *bc*_1_ complex [66,72].

**Figure 6:**
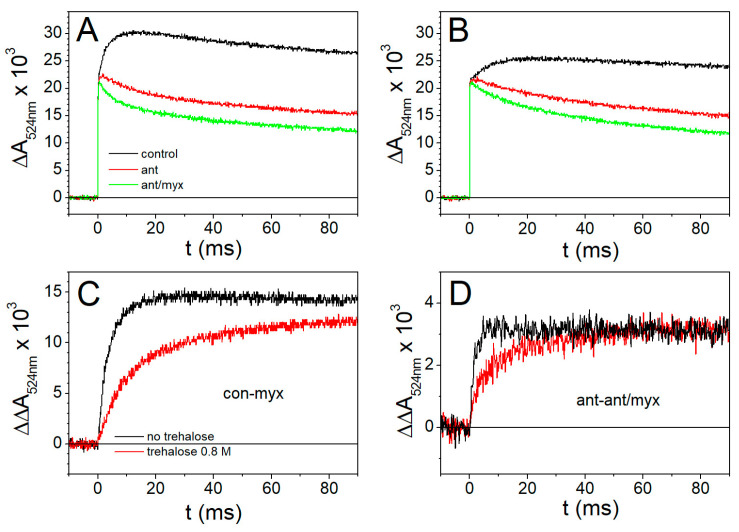
Kinetics of the electrogenic reactions along the low-potential cyt *b* chain of the cyt *bc*_1_ complex. Flash-induced carotenoid shift recorded in the absence (panel **A**) or in the presence of trehalose 0.8 M (panel **B**). In panels A and B, black traces were acquired in the absence of inhibitors; red traces in the presence of 10 μM antimycin; and green traces with 10 μM antimycin plus 2 μM myxothiazol. Panel (**C**) shows the difference between traces without inhibitors and traces in the presence of both antimycin and myxothiazol (black–green traces in panels **A**,**B**), and in the absence (black) or in the presence (red) of 0.8 M trehalose. Panel (**D**) presents the difference between traces recorded in the presence of antimycin and those acquired in the presence of antimycin plus myxothiazol (red–green traces in panels **A**,**B**) in the absence (black) or in the presence (red) of 0.8 M trehalose. See Section 4.3 for other experimental details. Traces are the average of 16 individual signals. A minimum dark time of 60 s was allowed between repetitive photoexcitation during averaging.

In Figure 6A,B, the time evolution of the flash-induced CS in the absence of inhibitors (black), in the presence of antimycin (red), and in the presence of antimycin plus myxothiazol (green) is shown for cromatophores suspended in buffer without trehalose (panel A) or in the presence of 0.8 M trehalose (panel B). In panel C, the difference between traces recorded in the absence of inhibitors and in the presence of antimycin plus myxothiazol (i.e., the antimycin-sensitive portion plus the myxothiazol-sensitive portion of the CS phase III) indicates, as expected, a strong slowdown of the kinetics in the presence of 0.8 M trehalose. The contribution of the antimycin-insensitive myxothiazol-sensitive phase, obtained by subtracting the signal recorded after the addition of myxothiazol from that recorded in the presence of antimycin alone, is shown in panel D. The kinetics of this myxothiazol-sensitive but antimycin-insensitive portion of CS phase III, which corresponds to electron transfer from cyt b_L_ to cyt b_H_, parallel the kinetics observed for the reduction in heme b_H_ in antimycin-inhibited chromatophores, both in the absence and in the presence of trehalose (see Section 2.2.2, Figure 5A). The half-times of this CS phase, estimated from the traces of Figure 6D, approximately 1.1 ms and 4.6 ms in the absence (black trace) and presence (red trace) of 0.8 M trehalose, are, in fact, in good agreement with those of cyt b_H_ reduction.

The results presented in Section 2.2.2 and Section 2.2.3 demonstrate that trehalose, at a high concentration, interferes with the oxidation of the quinone at the Q_o_ site, significantly slowing down the electron transfer along the cyt b chain of the complex. The quinol oxidation reaction at the Q_o_ site occurs in a concerted manner. The first electron is donated to the iron–sulfur center, generating semiquinone at the site. The semiquinone has a redox potential low enough to reduce heme b_L_ and oxidizes to quinone, thus delivering the second electron onto the low-potential arm formed by heme b_L_ and b_H_ of the *bc*_1_ complex [60]. The slowdown induced by trehalose in the reduction in cyt b_H_ can, therefore, be due to the inhibition of semiquinone oxidation (transfer of the second electron) or to an impairment of the transfer of the first electron from QH_2_ along the high-potential chain of the complex, which sequentially reduces the Fe_2_S_2_ center and cyt *c*_1_. The consequent formation of the semiquinone at Q_o_ is, in fact, a necessary condition for the transfer of the second electron to heme b_L_ (see Section 1).

In order to disentangle between these possibilities, we studied the kinetics of flash-induced cyt (*c*_1_ *+ c*_2_) redox changes, which can be directly monitored spectrophotometrically at 551–542 nm.

#### 2.2.4. Effect of High-Concentration Trehalose on Flash-Induced Redox Changes in Cytochromes c

**Figure 7:**
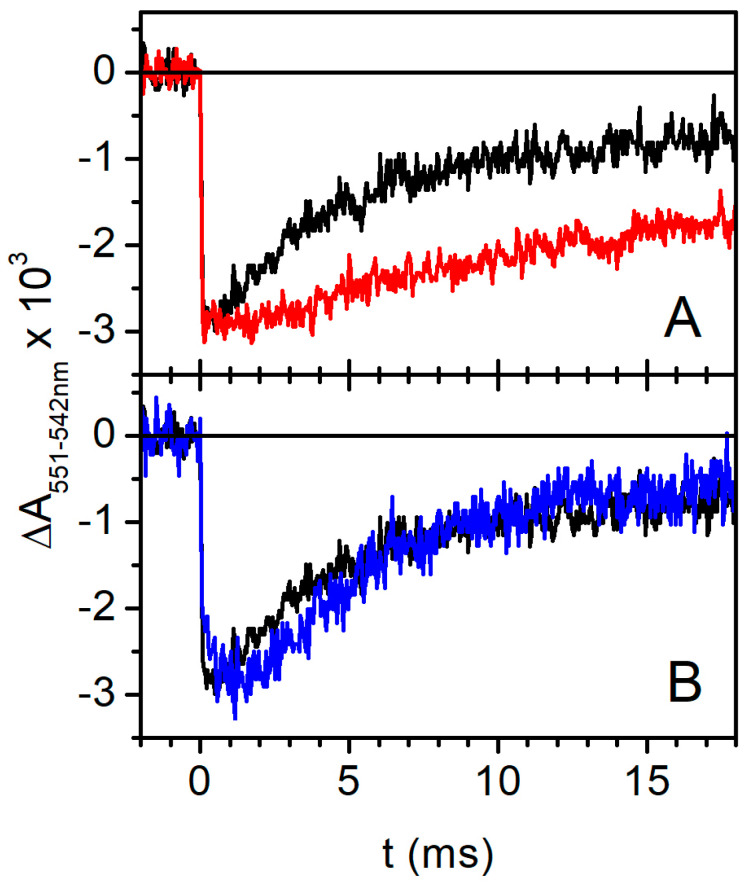
Kinetics of flash-induced cytochromes c redox changes. Panel (**A**): oxidation/re-reduction kinetics of cytochromes c in the absence (black trace) and in the presence (red trace) of 0.8 M trehalose. Panel (**B**): oxidation/re-reduction kinetics of cytochromes c in the absence (black trace) and in the presence of 35% (*w*/*w*) glycerol (blue trace). See Section 4.3 for additional experimental details. Traces are the average of 16 individual signals for each of the two wavelengths. A minimum dark time of 60 s was allowed between repetitive photoexcitation during averaging.

Figure 7A shows the time course of flash-induced cyt (*c*_1_ + *c*_2_) redox change, i.e., the unresolved photooxidation and subsequent re-reduction in cyt (*c*_1_ + *c*_2_) in the millisecond time range in the absence (black trace) and in the presence of 0.8 M trehalose (red trace). The addition of trehalose strongly reduced the rate of the re-reduction in cytochromes *c*, implying that the transfer of the first electron provided by the oxidation of quinol to semiquinone at the Q_o_ site is strongly inhibited by trehalose. It has to be noted that, under the reducing conditions of our experiment, in the dark-adapted chromatophore, all the components of the high-potential chain, i.e., soluble cyt *c*_2_, cyt *c*_1_, and the Rieske Fe_2_S_2_ center, are reduced before flash photoexcitation. The oxidation of quinol to semiquinone at the Q_o_ site requires, therefore, that the oxidizing equivalent generated by the photooxidation of the primary donor P of the RC be transferred by the soluble cyt *c*_2_ to the high-potential chain of the complex, sequentially oxidizing cyt *c*_1_ and the Fe_2_S_2_ center. Since this electron transfer chain involves a large-scale movement of the globular portion of the ISP from the proximity of the Q_o_ site to the site of interaction with cyt c_1_ and the shuttling of cyt *c*_2_ between the cyt *c*_1_ and the RC, the increase in viscosity induced by 0.8 M trehalose could, in principle, influence the rate of electron transfer mediated by these mobile redox partners (see Figure 1) and be, consequently, responsible for the slowdown observed in the re-reduction in cyt (*c*_1_ + *c*_2_).

In order to test this possibility, we studied the oxidation and subsequent re-reduction in cyt (*c*_1_ + *c*_2_) in chromatophores incubated in the presence of 35% (*w*/*w*) glycerol, i.e., a glycerol concentration that imposes a viscosity similar to that imposed by 0.8 M trehalose [73].

As highlighted in Figure 7B’s blue trace, the re-reduction kinetics of cyt (*c*_1_ + *c*_2_) photooxidized following a single flash is not significantly affected by the addition of glycerol, as compared to the control (chromatophores in buffer, black trace). Since this result excludes that the slowdown induced by trehalose on the re-reduction in oxidized cyt (*c*_1_ + *c*_2_) is a mere effect of increased viscosity, it can, in principle, result from (i) a slowed interaction of soluble *c*_2_ with cyt *c*_1_ of the *bc*_1_ complex; (ii) an impaired oxidation of the Fe_2_S_2_ center by cyt *c*_1_; or (iii) a slowed re-reduction in oxidized Fe_2_S_2_ by quinol at the Q_o_ site. Disentanglement between these possibilities can be achieved by examining the effects of specific inhibitors of the *bc*_1_ complex that bind in close proximity of the Q_o_ site on the re-reduction kinetics of cyt (*c*_1_ + *c*_2_) after a flash.

#### 2.2.5. Effect of Qo Site Inhibitors on the Re-Reduction in Photooxidized Cytochromes c

**Figure 8:**
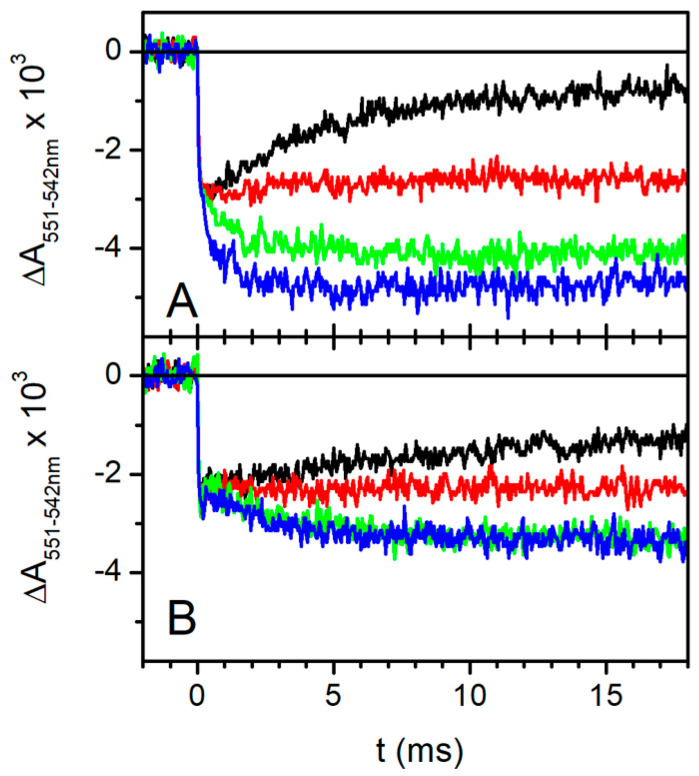
Kinetics of cytochromes *c* redox changes following a single turnover flash in the absence (panel **A**) and in the presence (panel **B**) of 0.8 M trehalose. Kinetics recorded in uninhibited chromatophores (black traces), in the presence of 10 μM antimycin (red traces), of 10 μM antimycin plus 2 μM myxothiazol (green traces), and in the presence of 2 μM stigmatellin plus 10 μM antimycin (blue traces). Traces are the average of 16 individual signals for each of the two wavelengths. A minimum dark time of 60 s was allowed between repetitive photoexcitation during averaging.

Figure 8 shows the effect of different inhibitors of the cyt *bc*_1_ complex, i.e., antimycin, myxothiazol, and stigmatellin, on the flash-induced redox changes in cyt (*c*_1_ + *c*_2_) in the absence (panel A) and in the presence (panel B) of 0.8 M trehalose. As already shown in Figure 7, without inhibitors, trehalose strongly slowed down the re-reduction in cytochromes *c*, which follows their fast (unresolved) oxidation upon flash excitation (black traces in panel A and B of Figure 8). After the addition of antimycin, the re-reduction in cyt (*c*_1_ + *c*_2_) is significantly slowed down both in the absence and presence of trehalose (Figure 8, red traces). Such an effect can be explained by considering that, according to the Q-cycle mechanism, when the semiquinone at the Q_i_ site is reduced to QH_2_ through the cyt *b* chain, further-reducing equivalents become available at the Q_o_ site in the absence of antimycin, which can contribute to cyt c re-reduction. The contribution of these additional reducing equivalents is, of course, suppressed in the presence of antimycin, which blocks electron transfer from heme b_H_ to the Q_i_ site, resulting in a marked inhibition of cyt (*c*_1_ *+ c*_2_) re-reduction. The occurrence of multiple oxidation turnovers of QH_2_ is expected in general to be more relevant at low cyt bc_1_/RC stoichiometris, a typical condition of the photosynthetic membranes of wild-type *Cba. sphaeroides* grown in the light and harvested in the stationary phase (see Section 4.1).

Myxothiazol, due to its complete inhibitory effect on the electron transfer from the Q_o_ site to the Fe_2_S_2_ center, totally abolishes the delivery of electrons coming from the Q_o_ site to the cyt (*c*_1_ + *c*_2_), which now receive electrons only from the Fe_2_S_2_ center. Consistently, myxothiazol causes an additional inhibition of the observed cyt (*c*_1_ + *c*_2_) re-reduction, even resulting in a significant stimulation of cyt (*c*_1_ + *c*_2_) oxidation amplitude after the flash, both in the absence and in the presence of trehalose (Figure 8, green traces in panels A and B).

In the presence of the inhibitor stigmatellin, the extrinsic portion of the ISP that contains the Fe_2_S_2_ center is blocked near the Q_o_ site, preventing the large-scale movement required for reduction by the Fe_2_S_2_ center of the oxidized cyt *c*_1_ [74]. As shown in Figure 8A, in the absence of trehalose, the addition of stigmatellin induces, as expected, a further stimulation of the oxidation amplitude of cyt (*c*_1_ + *c*_2_), preventing their re-reduction by the Fe_2_S_2_ center (Figure 8A, blue trace). Interestingly, in the presence of 0.8 M trehalose, the addition of stigmatellin does not stimulate the oxidation amplitude of the cyt (*c*_1_ + *c*_2_) (Figure 8B, blue trace), indicating that, even in the absence of the inhibitor, the electron transfer from the Fe_2_S_2_ center to cyt *c*_1_ is already strongly impaired in the presence of trehalose. We infer that the effects of trehalose on the electron transfer reactions catalyzed by the cyt *bc*_1_ complex originate from their interaction with the extrinsic domain of the ISP. When considering that the globular portion of the ISP faces the lumen of the chromatophores (see Figure 1), this result suggests that trehalose at a high concentration is able to permeate across the chromatophores membrane and hinder the movement of the ISP.

#### 2.2.6. Evaluation of Trehalose Content in Chromatophores

In order to ascertain the ability of trehalose to permeate the chromatophore membrane, we used a direct biochemical approach to detect the presence of trehalose in the chromatophore lumen following the incubation of chromatophores in the presence of externally added trehalose. To this end, an aliquot of chromatophores corresponding to a total bacteriochlorophyll concentration of 2 mM was diluted 1:1 with a solution of 1.6 M trehalose in 25 mM Hepes, pH 7.5, and incubated in the dark at room temperature. A total of 1 mM KCN was added to the chromatophore/trehalose mixture to block any oxidizing activity during the incubation time. At defined time intervals, a 500 μL volume of the mixture was taken and frozen at −20 °C. The integrity of the chromatophores after 3 h of incubation in 0.8 M trehalose was verified by recording the kinetics of flash-induced CS, which was found unchanged as compared to that measured in chromatophores incubated in buffer without trehalose (see Figure S1, Section S1, Supplementary Materials). The trehalose content in samples picked up at different times was determined by the anthrone method, described in detail in Section S2, Figure S2. In Figure S3, Section S3, the absorption spectra of the samples obtained from the trehalose-incubated chromatophores suspensions after the anthrone-trehalose colorimetric reaction are shown. Spectra were corrected for contributions from the zero time sample, i.e., a sample withdrawn immediately after trehalose addition (see protocol in Section S2 of the Supplementary Materials).

**Figure 9:**
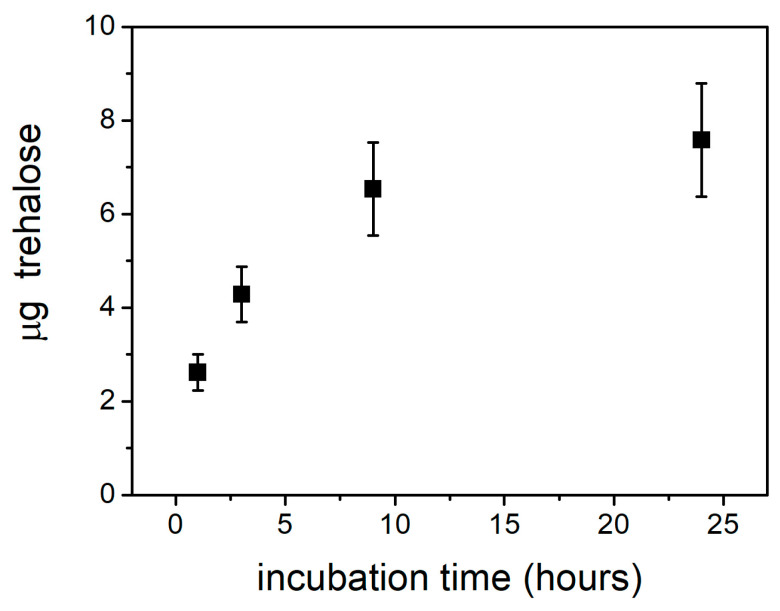
Mass of trehalose content of chromatophores as a function of incubation time in the presence of externally added 0.8 M trehalose. See Section 4.4 and Sections S2 and S3 of the Supplementary Materials for experimental details.

Figure 9 shows the trehalose mass content of chromatophores as a function of the time of incubation in 0.8 M trehalose. The increase over time of the trehalose content of chromatophores highlights an asymptotic trend, where 50% of the maximum content of trehalose is reached in about 3 h of incubation. Although the amount of trehalose present inside the chromatophore vesicles determined by this approach does not translate easily into values of internal trehalose concentrations (see Section 3), these data clearly show that trehalose added externally at high concentration permeates the chromatophore membrane with slow kinetics.

## 3. Discussion

The joint use of electrometric and spectrophotometric approaches allowed us to add new information on the interaction of the disaccharide trehalose with integral membrane complexes that catalyze photoactivated electron/proton transfer processes within the energy transducing membrane of photosynthetic bacteria. We focused on the membrane protein complexes, the RC, and the cyt *bc*_1_ complex, which compose the electron transfer chain of the photosynthetic bacterium *Cereibacter spaheroides*, as studied in their native lipid environment, i.e., in vesicles (chromatophores) isolated from the intracytoplasmic membrane. This simple photosynthetic system represents a model system particularly suited to study trehalose-induced effects, having been extensively characterized from the structural and functional point of view. In fact, the crystallographic structures of the RC and of the cyt *bc*_1_ complex have been determined to atomic resolution [75,76] and the thermodynamics and kinetics of the partial electron/proton transfer reactions occurring inside the complexes are known in great detail (for a review, see [77]).

All the experimental studies and molecular dynamics simulations aimed to elucidate protein–trehalose interaction in liquid ternary water–trehalose–protein systems have been performed on soluble proteins. However, they can also provide a useful basis to discuss the interaction of trehalose with integral membrane proteins such as the RC and the cyt *bc*_1_ complex embedded into the chromatophore membrane. Both these membrane protein complexes have, in fact, portions facing the water compartments and, additionally, include hydrophilic water-exposed domains: in the case of the RC, the globular H-subunit, which caps the Q_B_ site, and the periplasmic RC surface, which docks the soluble cyt *c*_2_; in the case of the cyt *bc*_1_ complex, this involves the extrinsic domain of the ISP and the hydrophilic portion of cyt *c*_1_, which protrudes into the lumen and hosts the reductase site for cyt *c*_2_. It is, therefore, important to understand how trehalose can be expected to interact with these water-exposed portions of the integral membrane complexes, on the basis of what is known from the study of its interaction with globular proteins in liquid solutions.

As mentioned in the Introduction, the models proposed to elucidate protein–trehalose interaction can be traced back essentially to two hypotheses: the water replacement hypothesis [11,16] and the water entrapment hypothesis [20,78,79,80]. Different sets of experimental and molecular dynamics data have been taken to support the former (see, e.g., [16,81]) or the latter [82,83,84,85,86] models. A recent structural and dynamical investigation of aqueous trehalose and sucrose myoglobin solutions based on neutron and X-ray diffraction and quasi-elastic neutron scattering [7] has put in evidence that the water replacement and water entrapment hypotheses should not be considered as mutually exclusive, but rather as simplified reference modes of trehalose–protein interactions that may coexist. In line with the notion that both modes of trehalose interaction with the protein (water replacement and water entrapment) are likely to co-occur, a previous long-scale molecular dynamics simulation of lysozyme in aqueous trehalose solution (0.75 M) [87] indicated that trehalose molecules distribute non-uniformly over the protein surfaces forming patches, mostly located around the less structured elements of the protein, intercalated by large water clusters. Solvent-exposed amino acid residues adjacent to trehalose clusters exhibited significantly reduced mobility.

In the following, keeping in mind the features of the trehalose–protein interaction outlined above, we will discuss the possible mechanisms underlying the observed effects of trehalose on the kinetics of Q_B_ protonation and cyt *c*_2_ oxidation within the RC and on the electron transfer processes catalyzed by the cyt *bc*_1_ complex.

As to the possible effects of trehalose on charge-separating processes catalyzed by the RC, we expected that they most likely concerned reactions occurring at the protein–solution interface or in the proximity of protein regions exposed to the solvent and, consequently, to trehalose. These processes are the oxidation of soluble cyt *c*_2_ by the photooxidized primary donor P^+^, occurring at the RC–lumen interface (see Figure 1) and the protonation of the doubly reduced quinone bound at the Q_B_ site, on the opposite side of the RC complex.

The first process consists of a first-order electron donation taking place within a preformed RC-cyt *c*_2_ complex (for a review see [88]) and a bimolecular collisional reaction due to the reaction of unbound cytochrome in solution.

The full reduction in the quinone at Q_B_ to quinol requires two subsequent photoexcitations: the first electron delivered by the primary photoreduced acceptor Q_A_^−^ to Q_B_ is not associated with the direct protonation of the quinone, while the second electron transfer from Q_A_^−^ to Q_B_^−^ involves the uptake of the first proton to form Q_B_H^−^; following this proton-coupled electron transfer step, the second proton is sequentially bound, resulting in Q_B_H_2_ formation [77].

As shown in Figure 2A,B, under conditions in which cyt *c*_2_ is partially reduced before photoexcitation and the ubiquinone pool is mostly oxidized, the electrometric signal recorded in dark-adapted chromatophore adsorbed to the phospholipid collodion membrane after the second flash exhibits a rise in the kinetic component, which is not observed after the first flash. The analysis of the difference between the signals acquired after the second and the first photoexcitation (Figure 2C) reveals two exponential phases characterized, in the absence of trehalose, by lifetimes τ_1_ ≅ 180 μs and τ_2_ ≅ 11 ms. In the presence of 0.8 M trehalose, the lifetimes of the two phases increase to τ_1_ ≅ 220 μs and τ_2_ ≅ 14 ms. The slower kinetic component, ascribed to electrogenic processes catalyzed by the cyt *bc*_1_ complex [57], appears to be slowed down by the addition of trehalose; however, as can be seen from Figure 2A,B, this phase is probably interfering with the decay of the electrometric signal, particularly in the presence of trehalose, which leads to a perceptible acceleration of the slower component. We are, therefore, reluctant to consider as quantitative the observed slowdown of the slower component.

The effects of trehalose on the phases of Δψ generation reflecting electrogenic events within the cyt *bc*_1_ complex have been better investigated by spectrophotometrically monitoring the carotenoid shift signals in chromatophore suspensions (see below for a discussion of these effects). The faster kinetic phase reflects the contribution to Δψ generation due to proton transfer from the external aqueous phase to the Q_B_ site of the RC leading to the formation of quinol after the second photoexcitation [57]. Its lifetime (about 180 μs in the absence of trehalose, in agreement with previous measurements [57]) increases to 220 μs upon the addition of 0.8 M trehalose, demonstrating a slowdown in the kinetics of Q_B_ protonation.

The Q_B_ site is deeply buried in the protein and the double protonation of quinone bound at Q_B_, which follows the delivery of the second electron and requires the long-range transfer of protons through the protein dielectric. This is accomplished by a chain of proton donors and acceptor groups (aminoacidic residues and bound water molecules), linked by hydrogen bonds and connecting the exterior aqueous phase to the Q_B_ site, along which protons are passed via the Grotthuss mechanism [89]. The pathways for the transfer of the first and second protons have been deeply and extensively investigated based on high-resolution crystal structures, site-directed mutagenesis, and metal ion binding (for a review, see [77]). A series of structural and kinetic studies performed in the presence of the proton transfer inhibitors Cd^2+^ and Zn^2+^ [90,91,92] have led to the tentative identification of dominant pathways which start from the RC surface and end on the carbonyls of the Q_B_ molecule. Two histidines (His-H126 and His-H128, both belonging to the H-subunit) appear to play a special role in collecting protons at the entry points of the pathways. It has been proposed that the attraction, retainment, and transfer of protons from the RC surface to internal groups is facilitated by surface acidic groups like carboxylates and histidines forming a type of “proton-collecting-antennas”. As an alternative to the well-defined dominant pathways mentioned above, delocalized models, considering multiple pathways acting in parallel, have been also suggested based on a different interpretation of the inhibitory transition metal effects [93,94]. A proton transfer to Q_B_ mediated by a large tangled hydrogen bond network has also been supported by conservation and hydrogen bond analysis [95] and by a recent molecular dynamics network analysis [96]. The latter study has identified three entries from clusters of surface residues located around His-H126, Glu-H224, and His-H68.

In view of the studies summarized above, and of the current models describing the trehalose–protein interaction in a solution, we propose that the slowdown of Q_B_ protonation observed by means of the electrometric approach in the presence of trehalose (Figure 2) is due to the perturbation introduced by the sugar on the hydrogen bond organization of the RC hydration layer in the regions exposed to the external water phase and involved in the entry of protons funneled to the Q_B_ site. It is important to point out that, according to the previous near-field scanning optical microscopy of chromatophores fused with lipid-impregnated collodion films, the average thickness of chromatophores attached to the membrane is ~0.03 µm, which is comparable to one or a few layers of chromatophores [97]. This implies that the reaction center residues involved in the protonation of quinone molecules bound at the Q_B_ site are exposed to trehalose dissolved in the external solution (see Figure 1).

According to the current models of trehalose–protein interaction, trehalose can hinder the entrance of protons into the proton transfer pathways, either by direct hydrogen binding to RC residues, which collect protons, or by stiffening the H-bond network of water molecules at the RC surface, involved in proton donation to residues forming the entry of the proton conducting pathways. We consider these possibilities not mutually exclusive since, in ternary trehalose–protein–water systems, the protein solvation layer most likely includes both clusters of trehalose molecules, H-bonded to exposed residues, and patches of the protein hydration shell, the dynamics of which is significantly slowed down by H-bonding with trehalose [21,87].

The electrometric measurements presented in Figure 3 were performed under conditions in which, upon flash excitation, only the primary charge-separated state P^+^Q_A_^−^ can be formed (within approximately 200 ps), followed by the oxidation of cyt *c*_2_, which donates the electron to P^+^. When cyt *c*_2_ is mostly reduced before the flash (green trace in Figure 3), the unresolved Δψ generation coupled to P^+^Q_A_^−^ formation is followed by a rise phase characterized by a lifetime τ ≅ 290 μs, ascribed to the electrogenic cyt *c*_2_ oxidation [57]. Upon incubation in the presence of 0.8 M trehalose, the latter phase (red trace in Figure 3) exhibits the same amplitude observed in the absence of the sugar; also, its kinetics are marginally affected (lifetime τ ≅ 330 μs), indicating that trehalose has a very limited, if any, effect on the rate of electron transfer to P^+^. We conclude that trehalose does not significantly perturb the structure and dielectric properties of the cyt *c*_2_-RC complex.

We switch now to discuss the effects observed on electron transfer reactions catalyzed by the cyt *bc*_1_ complex. The effects observed in the presence of 0.8 M trehalose on the antimycin-sensitive (Figure 4) and on the antimycin-insensitive but myxothiazol-sensitive (Figure 6) phase III of the carotenoid shift indicate clearly that Δψ generation, due to vectorial electron transfer between heme b_L_ and b_H_ as well as proton uptake at the Q_i_ site, is markedly slowed down by trehalose. In quantitative agreement with these observations, the direct monitoring of the kinetics of heme b_H_ reduction in the presence of antimycin (Figure 5A) confirmed that the transfer of the electron released upon the oxidation of QH_2_ at the Q_o_ site of the complex along the cyt *b* chain (see Figure 1) is significantly retarded by trehalose. In view of the slowdown observed in the antimycin-sensitive phase III of the carotenoid shift, ascribed to the electrogenic protonation of quinone bound at the Q_i_ site, coupled to electron transfer from heme b_H_, we have examined the possibility that trehalose affected the catalytic activity of the Q_i_ site. This can be achieved by studying the kinetics of the reversed electron transfer from QH_2_ reacting at the Q_i_ site to heme b_H_ [69]. The pH dependence of the redox potential of the Qpool and of the heme b_H_ make it, in fact, possible to observe, at alkaline pH, with the Qpool fully oxidized, and in the presence of the inhibitor myxothiazol which blocks the Q_o_ site, the reduction in heme b_H_ sensitive to antimycin, i.e., proceeding through the Q_i_ site by the oxidation of QH_2_ released by the Q_B_ site of the RC [69]. The kinetics of this reaction (Figure 5B) was found unaffected by 0.8 M trehalose, implying that the slowdown observed in electron transfer through the cyt *b* chain was not due to an effect of trehalose on the Q_i_ site, despite its location on the external side of the chromatophore membrane.

Under reducing conditions, the components of the high-potential branch (Fe_2_S_2_ and cyt c_1_) are reduced in dark-adapted chromatophore before the flash; therefore, according to the Q-cycle mechanism [60,61], a prerequisite to observe the reduction in cyts *b* and the associated Δψ generation phases is that, following the flash, the primary photooxidized donor P^+^ of the RC oxidizes in sequence the soluble cyt *c*_2_, cyt *c*_1_, and Fe_2_S_2_ center, which, being reduced by QH_2_ at the Q_o_ site, will generate the semiquinone which, in turn, reduces cyts *b*. As a consequence, a slowdown in the electron transfer processes along the high-redox-potential branch of the complex would result in a corresponding slowdown of electron transfer along the cyt *b* branch, as we observed in the presence of highly concentrated trehalose. This consideration prompted us to examine the kinetics of cyt(*c*_1_ + *c*_2_) re-reduction following their flash-induced oxidation (Figure 7A), which came out to be strongly inhibited in the presence of 0.8 M trehalose. We can exclude that such an effect is due to an increase in the viscosity because, in the presence of glycerol at a concentration which leads to the same medium viscosity increase induced by the addition of trehalose, no effect could be detected on the re-reduction kinetics of cyt (*c*_1_ + *c*_2_) (Figure 7B). The kinetics of the flash-induced redox changes in cyt (*c*_1_ + *c*_2_) in the presence of myxothazol and stigmatellin (Figure 8) allows us, in principle, to localize more precisely the inhibitory effect of trehalose. Myxothiazol, which blocks electron transfer from the Q_o_ site to the oxidized Fe_2_S_2_, strongly inhibits the re-reduction in cyt (*c*_1_ + *c*_2_), even stimulating the oxidation extent after the flash both in the absence and in the presence of trehalose. As expected, in the absence of trehalose, stigmatellin further increases the extent of cyt (*c*_1_ + *c*_2_) oxidation upon photoexcitation (Figure 8A); this inhibitor, in fact, blocks electron transfer from the Fe_2_S_2_ center of the ISP to cyt *c*_1_, thus further reducing the flow of electrons to cyt (*c*_1_ + *c*_2_). Remarkably, in the presence of trehalose, stigmatellin has no additional effect on the cyt (*c*_1_ + *c*_2_) redox changes as compared to what was observed in the presence of myxothiazol (Figure 8B). This finding strongly suggests that, in the presence of trehalose, the oxidation of the Fe_2_S_2_ center by cyt *c*_1_ is already strongly impaired, even in the absence of stigmatellin. We cannot, in principle, exclude that, besides affecting this electron transfer step, trehalose causes, in addition, a slowdown of cyt *c*_1_ oxidation by perturbing the interaction between the soluble cyt *c*_2_ oxidized by the RC with the cyt *c*_1_. However, we consider this additional possibility unlikely based on the fact that, as revealed by the electrometric response (Figure 3), trehalose does not significantly affect the oxidation of cyt *c*_2_ by the RC. This process, similar to the oxidation of cyt *c*_1_, involves the formation of a transitory reaction complex between the soluble cyt *c*_2_ and the hydrophilic portion of an integral protein facing the lumen of the chromatophore membrane. In conclusion, the analysis of the effects of trehalose on electron/proton transfer processes catalyzed by the cyt *bc*_1_ complex localizes its predominant action to an electron transfer step involving the ISP and cyt *c*_1_.

Various high-resolution crystal structures of the cyt *bc*_1_ complex have revealed that the extrinsic domain of the ISP, containing the Fe_2_S_2_ center, occupies one of two different conformations within the complex: in one position, the Fe_2_S_2_ center is close enough to the heme group of cyt *c*_1_ to allow for the transfer of the electron from the Fe_2_S_2_ cluster to cyt *c*_1_, while, in the other, the Fe_2_S_2_ center is closer to the Q_o_ site, from which it receives electrons provided by QH_2_ oxidation. Since neither position is compatible with both electron tunneling reactions proceeding at the appropriate rate, it was inferred that catalysis at the Q_o_ site required a large-scale movement of the extrinsic ISP domain [75,98]. Subsequently, a large body of spectroscopic, kinetic, and biochemical data have definitively established that the movement of the extrinsic domain of the ISP is essential to shuttle electrons between the Q_o_ site and the cyt *c*_1_ heme [98].

**Figure 10:**
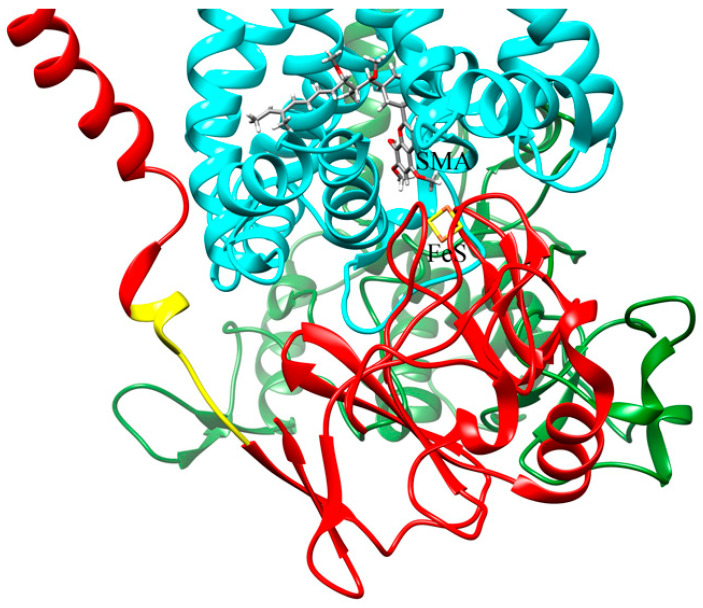
Structure of the *Cba. sphaeroides* cyt *bc*_1_ complex inhibited by stigmatellin (PDB entry: 6NIN). For the sake of clarity, only one monomer of the dimeric cyt *bc*_1_ complex is presented. The cytochrome *b*, ISP, and cytochrome *c*_1_ subunit are presented in cyan, red, and green, respectively. The ISP hinge region is highlighted in yellow. SMA, stigmatellin; FeS, Fe_2_S_2_ iron sulfur cluster of the ISP.

Certain structural features of the ISP are important in understanding the possible modes of the trehalose interaction responsible for the observed impairment in electron transfer from the Fe_2_S_2_ center to cyt *c*_1_ heme. In the different structures, the membrane-anchoring region of the extrinsic ISP domain remains fixed, implying that the oscillation of the extrinsic domain containing the redox center between the Q_o_ and the cyt *c*_1_ positions requires conformational changes in the flexible region which connects the anchor transmembrane helix to the mobile domain (see Figure 10). This region, acting as a hinge, is, therefore, expected to play a pivotal role in the movement of the extrinsic ISP domain. Indeed, in *Rhodobacter capsulatus*, a bacterium very similar to *Cba. sphaeroides*, mutations in the hinge region of the ISP that prevent its conformational changes have been shown to completely inactivate catalysis in the *bc*_1_ complex [99].

Since several residues belonging to the hinge region of the ISP are exposed to the solvent in the lumen side of the chromatophores, we propose that trehalose interacts with this region either directly by forming H-bonds with the exposed residues or by promoting the formation of strong H-bonds with trapped water molecules.

In both interaction modalities, trehalose can be expected to strongly hamper the movement of the extrinsic ISP domain and, consequently, severely slow down the oxidation of the Fe_2_S_2_ center by the cyt *c*_1_ heme. As it was mentioned above, molecular dynamics simulations of lysozyme in highly concentrated aqueous trehalose solution indicated that trehalose forms direct H-bonds with surface residues preferably around the less structured elements of the protein [87]; it is, therefore, conceivable that trehalose binds directly to residues of the hinge region of the extrinsic ISP domain, giving rise to trehalose patches, which stiffen the hinge affecting the oscillation of the ISP extrinsic domain. Molecular dynamics simulations of lysozyme [87] showed that residues near trehalose patches are strongly constrained and exhibit slowed dynamics. In this respect, our observation that 0.4M trehalose has a very limited, if any, effect on the processes catalyzed by the cyt *bc*_1_ complex as compared to 0.8M trehalose (see Figure 4 and Figure 5) could reflect a cooperative behavior attributable to the formation of trehalose clusters bound to the surface of the ISP extrinsic domain, occurring only above a sugar concentration threshold. Also, in the perspective of preferential hydration (trehalose exclusion or water entrapment hypothesis), the formation of a hydrogen bond network involving constrained water molecules of the hydration layer and residues of the hinge region are equally expected to slow down the movement of the Fe_2_S_2_ cluster domain. An alternative or concomitant possibility is that a similar direct or indirect H-bond interaction of trehalose occurs with exposed residues of the cyt *b* and/or cyt *c*_1_ subunit involved in contact with the extrinsic ISP domain during its movement. Molecular dynamics simulations of the rotation of the soluble ISP domain [100] suggest, in fact, that the movement is guided along its trajectory by several hydrogen bonds between exposed residues of the soluble head of ISP and residues of the cyt *b* and *c*_1_ subunits, undergoing sequential breaking and formation during the rotation. A trehalose-induced perturbation of these H-bonds would result in severe impairment of the rotation, or in the non-optimal reciprocal positioning in the encounter of the extrinsic ISP domain with cyt *c*_1_ for electron transfer between the two redox partners.

It should be noted that the slowdown observed in the electron/proton transfer processes associated with QH_2_ oxidation at the Q_o_ site of the cyt *bc*_1_ complex in the presence of 0.8 M trehalose is essentially negligible at a sugar concentration of 0.4 M. The fact that a high concentration of trehalose is needed to inhibit the oxidation of the Fe_2_S_2_ center following photoexcitation may be related to the observation that trehalose significantly disrupts the tetrahedral network of water molecules by ordering the water molecules around itself and forming its own hydrogen-bonded network only above a threshold concentration (30% in weight, i.e., about 0.4 M) [12,101].

It is possible, although less probable, that a more delocalized interaction of trehalose with the water-exposed portions of the *bc*_1_, both on the external and on the lumenal side of chromatophore, contribute to the determination of the kinetic effects induced by trehalose on electron transfer due to an overall perturbation of the structure of the protein complex. Trehalose-induced structural perturbations have been observed in a model globular protein (bovine serum albumin), resulting in protein compaction and increased α-helicity [102].

Finally, it has to be considered that trehalose also interacts with phospholipid bilayers, increasing their stability at elevated temperatures and low hydration, and decreasing the gel-to-liquid crystalline phase transition temperature [103]. Most of the experimental and simulative studies support the view that trehalose interacts directly with the bilayer by forming hydrogen bonds with the lipid headgroups [104,105,106,107,108,109,110,111], although water is probably not completely expelled from the membrane surface [112,113,114]. Whatever the mechanism of the trehalose–lipid interaction, it is possible that the structure/dynamics of the lipid component of the chromatophore membrane and its hydration are influenced by trehalose, which may induce effects like the lateral expansion of the lipid headgroups associated with the intercalation of trehalose, the reduction of the thickness of the bilayer [114], and an increase in the order parameters of the acyl chains [115]. These alterations may, in turn, affect the conformation of RC and the *bc*_1_ complex within the membrane bilayer. We expect, however, that, if any of these indirect effects occur, their contributions be negligible, and also in view of the fact that the chromatophore membrane is formed predominantly by proteins and to a minor extent by phospholipids [116].

It is generally believed that the permeability of phospholipid bilayers to disaccharides and specifically to trehalose is very low, as would be expected for a polar molecule of a relatively large size. Despite this, we observed that trehalose, externally added at high concentrations, markedly affects electron transfer reactions catalyzed by QH_2_ oxidation at the Q_o_ site located close to the lumen side of chromatophores, and that, additionally, this effect most likely involves the dynamics of the extrinsic portion of the ISP hanging out on the internal compartment of the chromatophore vesicle. In principle, the above-mentioned trehalose-induced effects could be due to the interaction of trehalose with the externally exposed portion of the protein complex and/or with the outer leaflet of the membrane. These interactions could result in conformational transitions transmitted to the inner leaflet of the bilayer and to the inner side of the protein complex. We believe, however, that such an “indirect” or “mediated” interaction of trehalose with the protein complex should not contribute significantly. In fact, a pre-incubation of 2–3 h of the chromatophore suspension after the addition of trehalose (see Section 4.3) was necessary before starting kinetic measurements to detect the trehalose-induced effects we observed on partial reactions involving redox centers close to the inner side of the cyt *bc*_1_ complex. This observation strongly suggests that trehalose, on the time scale of a few hours, is able to permeate the chromatophore membrane and that the effects induced by trehalose are mainly attributable to its permeation within the chromatophores. This conclusion was corroborated by the estimation, through the anthrone method, of the amount of trehalose present in chromatophore suspensions, following incubation for different times in the presence of the sugar, after the washing of the vesicles. After correction for sugar detected in a chromatophore suspension, and immediately being washed after the addition of trehalose, the trehalose content of chromatophores was found to increase as a function of time (Figure 9), implying that, during the incubation time, the sugar permeates the chromatophore membrane. After about 3 h of incubation, half of the trehalose content corresponding to saturation is detected.

The determination of the trehalose internal concentration from the trehalose mass content within vesicles, as detected by the anthrone method, appears to be problematic because it would require a reliable value for the effective volume of the internal compartment of chromatophores, which could also be affected to some extent by osmotic effects.

As to the fact that trehalose, at variance with its expected behavior, slowly permeates the membrane, it is worth noting that the chromatophores can hardly be assimilated to pure phospholipid vesicles since their lipid content is very limited when compared to the total content of proteins that are largely predominant in the composition of the chromatophore membrane. In fact, according to the model developed by Singharoy et al. [116] based on atomic force microscopy data and computer simulations, the lipid component of the chromatophore membrane bilayer can be evaluated to occupy only approximately 16% of the total chromatophore surface. We cannot exclude that permeation is facilitated by a still unknown transmembrane protein transporter. It is possible that chromatophores contain such transporters, like how eucaryotic mitochondria contain a large number of transmembrane solute transporters, which could be not exactly substrate-specific [117]. Alternatively, the slow trehalose permeation could be controlled by lipid fluctuations, giving rise to transient defects in the bilayer. It should be mentioned that the *Cba. sphaeroides* chromatophores contain phosphatidyl-ethanolamine and cardiolipin [118,119,120], classified as non-bilayer-forming lipids, the presence of which is associated with the destabilization of the membrane structure [121].

The evidence that trehalose, at high concentrations, permeates the membrane of chromatophores suggests that other disaccharides may do so, making it possible to compare their effects on the electron/proton transfer processes with those examined in the presence of trehalose. Preliminary results obtained in the presence of 0.8M sucrose indicate that, similarly to what was observed in the presence of 0.8M trehalose, the onset kinetics of phase three of the carotenoid shift of chromatophores incubated in the presence of this structurally analog sugar is significantly slowed down (see Figure S4, Section S4). Interestingly, however, the slowing effect observed in the presence of sucrose was markedly less pronounced than that induced by trehalose. This observation suggests that sucrose affects the electron transfer processes catalyzed by the cyt *bc*_1_ complex similarly to trehalose, but less effectively, in agreement with the notion that sucrose perturbs, to a lesser extent, the intermolecular H-bond network of water [27,28,29,30,31], and is characterized by a weaker structural and dynamical interaction with proteins and their hydration layers [7,13,35]. Based on the observed effect of sucrose on the third phase of the carotenoid shift (Figure S4), experiments aimed at systematically comparing the response of partial electron transfer reactions to trehalose and sucrose are underway in our laboratories.

## 4. Materials and Methods

### 4.1. Bacterial Growth and Chromatophore Preparation

*Cereibacter sphaeroides* 2.4.1 cells were grown photosynthetically at 30 °C in Sistrom medium [122] and harvested in the stationary phase. Chromatophores were prepared as described in [50] using Hepes 25 mM (VWR Chemicals, Leuven, Belgium), pH 7.5, as a buffer, and kept at 4 °C for a maximum of four days before use. The total bacteriochlorophyll content in the chromatophore suspensions was measured by organic solvent extraction, as described in [123].

### 4.2. Direct Electrometrical Technique

Photoelectric responses of membrane vesicles (chromatophores) were measured electrometrically using a phospholipid-impregnated collodion film as described previously [54,55]. The film separated two electrolyte-containing compartments of a Teflon chamber. Chromatophore suspension was added into one of the compartments (final concentration of the bacteriochlorophyll ~50 μg/mL). The association of chromatophores with the collodion film was achieved upon 1.5–2 h incubation at room temperature in the presence of 20 mM MgCl_2_. Then, the excess of membrane vesicles not associated with the collodion film was removed by an appropriate incubation medium (20 mM Hepes, pH 7.5) without MgCl_2_ using a peristaltic pump. Ag/AgCl electrodes were used to measure Δψ across the membrane with adsorbed chromatophores. The voltage output was coupled via an operational amplifier (Burr Brown 3554BM, Gage Applied Technology, Schaumburg, IL, USA) to a CS8012 Gage and then to a computer.

To reconstruct the function of loosely bound Q_B_ and the membrane ubiquinone pool, CoQ_10_ was added to the lipid solution required for the impregnation of the collodion film (20 mg/mL) [56,63].

Light-dependent reactions in this system were induced by laser flashes (frequency-doubled YAG, 532 nm; pulse half-width, 15 ns; pulse energy, 15 mJ; Quantel, Les Ulis, France). This method allows for the direct measurement of undistorted electric signals with ~200 ns time resolution. Each photoelectricity measurement was repeated at least 3 times. All measurements were carried out at 23 ± 1 °C.

The kinetics of flash-induced voltage generation presented in Figure 2C were fitted to the sum of 2 exponential components using the scripts for Matlab R2023a [MATLAB: 9.14.0, The MathWorks Inc., Natick, MA, USA (2023) URL: https://www.mathworks.com/, accessed on 13 December 2024]

### 4.3. Time-Resolved Visible Absorption Spectroscopy

Spectrophotometric measurements were carried out on chromatophores resuspended at a concentration corresponding to 40 μM total bacteriochlorophyll in 25 mM Na-Hepes buffer, pH 7.5, 50 mM KCl, Na-succinate and Na-fumarate both at 2 mM concentration, and 1 mM KCN in an open unstirred cuvette [124]; 8 μM 1,2 Naphthoquinone and 1,4 Naphthoquinone, and 5 μM TMPD were added as redox mediators. The kinetics of heme b_H_ reduction through the Q_i_ site of *bc*_1_ complex was measured in 50 mM Na-CHES buffer, pH 9.5 [125]. The time evolution of the carotenoid band shift was elicited by a single xenon flash (duration of less of 8 μs), and redox changes induced in the heme b_H_ and in the cytochromes *c* (*c*_1_ + *c*_2_) were detected using an apparatus of local design as described in [126]. Spectrophotometric traces of the carotenoid shift, and of the redox changes in cytochromes *c* and heme b_H_, were recorded at 524 nm, 551–542 nm, and 561–569 nm, respectively [127]. The kinetics of redox changes in the heme *b*_H_ and cytochromes *c* were monitored in the presence of 10 μM valinomycin to rapidly collapse electrochromic spectral contribution interfering with the signal.

Each kinetic experiment was repeated several times by using totally independent samples and different chromatophore preparations. It is worth noting that, over a set of independent experiments, some variability in the kinetics of the third phase of the carotenoid shift and of the heme b_H_ reduction, present also in the control traces in the absence of trehalose, was systematically observed. This variability can result in a difference of half-times ranging from 10% to 25%. Such a variability is not surprising when considering that the observed kinetics, reflecting the oxidation of QH_2_ at the Q_o_ or Q_i_ sites of the cyt *bc*_1_ complex, are extremely sensitive to the redox state of the UQ pool. In order to establish an optimal redox poise, E_h_, of the system, we used the fumarate/succinate couple and a mixture of redox mediators (see above). This mixture should provide reducing conditions (E_h_ ≈ 90 mV) for which approximately half of the UQ pool is reduced. However, particularly in the presence of oxygen, we expect that the redox poise can vary from one sample to the other at least by 20 mV, i.e., E_h_ = (90 ± 20) mV. By using the Nernst equation, it can be easily concluded that this uncertainty of E_h_ would correspond to an uncertainty of 5 reduced UQ molecules in a pool formed by 30 UQ molecules per RC, i.e., in a number of reduced UQ molecule equal to 15 ± 5 per RC. Such a variability, given the collisional nature of UQH_2_ oxidation at the sites of the cyt *bc*_1_ complex, is expected to result in a considerable variability in the kinetics of phase three of the carotenoid shift and of heme b_H_ reduction, comparable to the one observed in our experiments.

When examining the effects of trehalose on the kinetics, after the addition of the sugar to the chromatophore suspension, samples were incubated for 3 h before starting signal acquisition. This pre-incubation was necessary to detect the trehalose-induced effects.

### 4.4. Trehalose Detection

The determination of the trehalose internal content of the chromatophores was performed with a colorimetric method based on the reaction of carbohydrates with anthrone in a highly acidic environment [128] following a cell extraction protocol described in [129] adapted to chromatophores. The construction of the calibration curve and the trehalose extraction protocol from the chromatophores are described in detail in Section S2, Figure S2 of the Supplementary Materials.

Briefly, a chromatophore suspension was diluted 1:1 with a 1.6 M trehalose solution. At defined times during incubation with trehalose, a 500 μL volume of this mixture was passed into a Sephadex G25 column (PD10 column, GE Healthcare, Uppsala, Sweden) that was pre-equilibrated with distilled water. The total bacteriochlorophyll content in the eluted bands, deprived of external trehalose, was carefully evaluated, and the chromatophores’ trehalose content determination was performed on a 200 μL volume brought to a bacteriochlorophyll concentration equal to 300 μM.

## Data Availability

The original contributions presented in this study are included in the article/Supplementary Material. Further inquiries can be directed to the corresponding author(s).

## Supplementary Materials

The following supporting information can be downloaded at: https://www.mdpi.com/article/10.3390/ijms252413420/s1.
