# Supplementary material for: Trehalose Interferes with the Photosynthetic Electron Transfer Chain of Cereibacter (Rhodobacter) sphaeroides Permeating the Bacterial Chromatophore Membrane
Source: Int J Mol Sci. 2024 Dec 14;25(24):13420. doi: 10.3390/ijms252413420 (PMC11678701; doi:10.3390/ijms252413420)
Supplement: Supplementary file 1 [file ijms-25-13420-s001.zip › ijms-3315971-supplementary.pdf]

Supplementary Materials

S1. Integrity of chromatophores exposed to 0.8M trehalose

To verify the integrity of the chromatophore membrane during the course of kinetic measurements, the carotenoid shift signal elicited by an actinic flash was recorded in the absence/presence of 10 µM valinomycin, after 3 hours of incubation in 0.8 M trehalose, plus the time necessary for kinetic mesurements of cyt b and cyt c redox changes, requiring the averaging of several signals (about 1 hour).

.

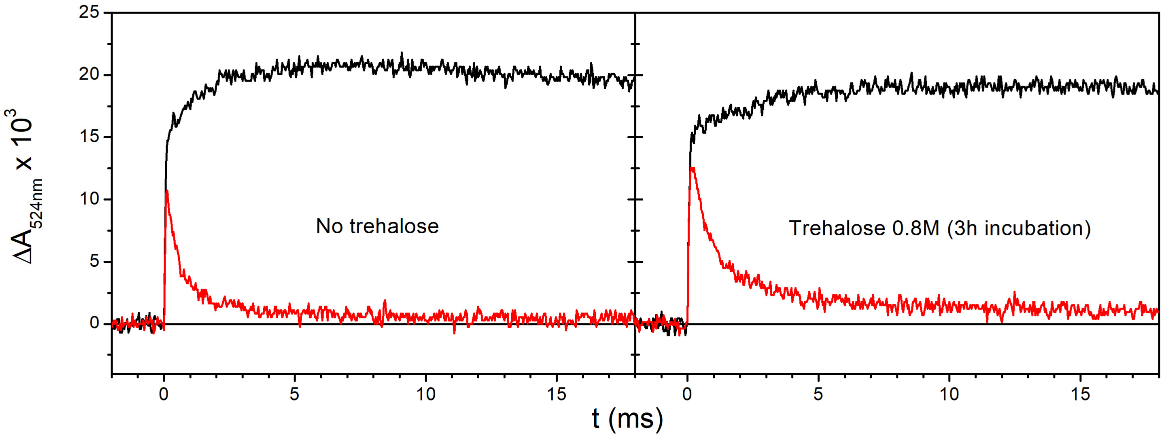

Figure S1. Left panel, control chromatophore sample without trehalose; right panel, chromatophores incubated in 0.8 M trehalose for 3 hours. Black traces, no ionophore; red traces, after addition of 10 µM valinomycin. The sample composition is described in section 4.3 of Materials and Methods. Traces are the average of 4 individual signals. A minimum dark time of 60 s was allowed between repetitive photoexcitation during averaging.

In the absence of trehalose (left panel) or after incubation in 0.8 M trehalose (right panel), chromatophores show a similar, typical carotenoid shift kinetics (black traces). In both samples, the addition of the ionophores valinomycin (red traces) induces a rapid collapse of the carotenoid shift signal due to the rapid decay of the flash-induced membrane potential. The amplitude of the carotenoid shift signal (black traces) does not undergo any significant change after the prolonged exposure to 0.8M trehalose; only the kinetics of the third phase is slowed down. This indicates that the trehalose induced effects observed cannot be attributed to shrinkage or collapse of the membrane vesicles due to osmotic effects, which would strongly affect the amplitude of the carotenoid shift signal, monitoring the onset of the membrane potential. These data indicate that chromatophores exposed for long times to 0.8 M trehalose maintain an intact membrane.

S2. Trehalose detection with the anthrone method

The aim was to detect by a direct method whether trehalose can permeate the chromatophore membrane.
Calibration curve. Standard samples of the desired mass of trehalose dissolved in 500 µL of water were prepared starting from a 1mg/mL trehalose stock solution and treated starting from point 14 of the protocol (see below).
Sample preparation. Aliquots of 500µL of chromatophores were taken at different incubation times in 0.8M trehalose and eluted by gravity through a Sephadex G25 gel filtration column (PD-10 Desalting column, GE Healthcare, exclusion limit of 5 KDa) pre-equilibrated with water. The band of chromatophores takes approximately 3 minutes to fully elute through the column's 5-centimeter Sephadex bed height.

Protocol for the detection of trehalose with the anthrone method.

1. Incubate the chromatophores in trehalose.
2. Elute an aliquot of chromatophores in a water-equilibrated Sephadex column at desired time point.
3. Evaluate the total BChl concentration in the chromatophore eluted band. Sample can be frozen if needed.
4. Add 4 volumes of methanol.
5. Warm for 1 hour in a thermostatic bath (or in a thermoblock) at 80 °C.
6. Separate membrane debris from solution by centrifugation.
7. Put the supernatant in a glass tube (1).
8. Resuspend the pellet in 80% methanol, 20% water.
9. Repeat step 6.
10. Collect the supernatant (2).
11. Merge the supernatants (1) and (2).
12. Evaporate the supernatant to obtain the fixed residue.
13. Resuspend the residue in water.
14. Gently add an equal volume of 95-98% sulfuric acid and mix slowly. The solution gets very hot.
15. Wait 30'.
16. Gently add a half volume of a solution with anthrone (4mg/mL anthrone in 95-98% sulfuric acid) and mix slowly.
17. Heat the samples at 100 °C for 5 minutes in a thermoblock.
18. Let them cool and measure the absorption spectrum from 500 to 700 nm.

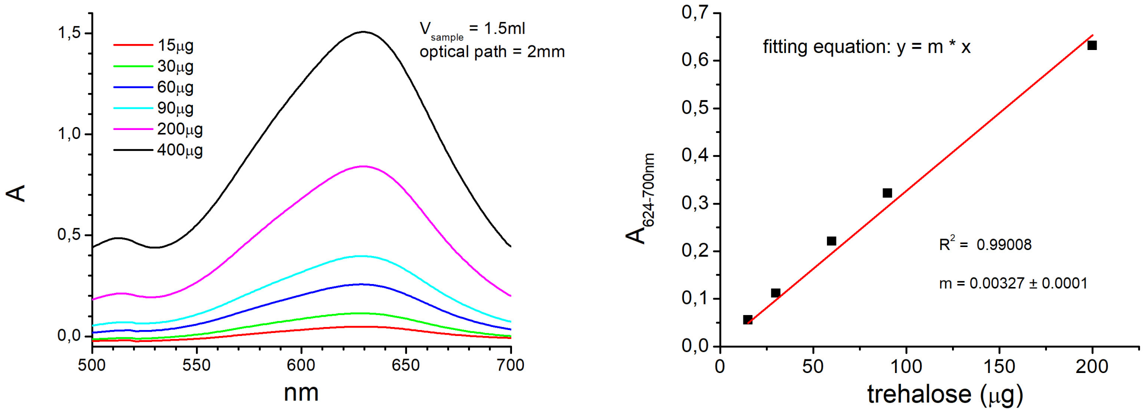

Figure S2. Left panel: absorption spectra of the standard samples. Right panel: calibration curve obtained from the A_{624}-$A_{700\ nm}$ values plotted against the µg of trehalose in the standard sample.

Judging from the linearity obtained, trehalose responds well to determination by the anthrone-based method. Note that the spectrum with the highest concentration of trehalose (400 µg) was not used for the linear regression of the calibration curve as it already absorbed too much, despite the spectra being recorded with cuvettes with an optical path reduced to 2 mm.

S3 Trehalose content in chromatophores

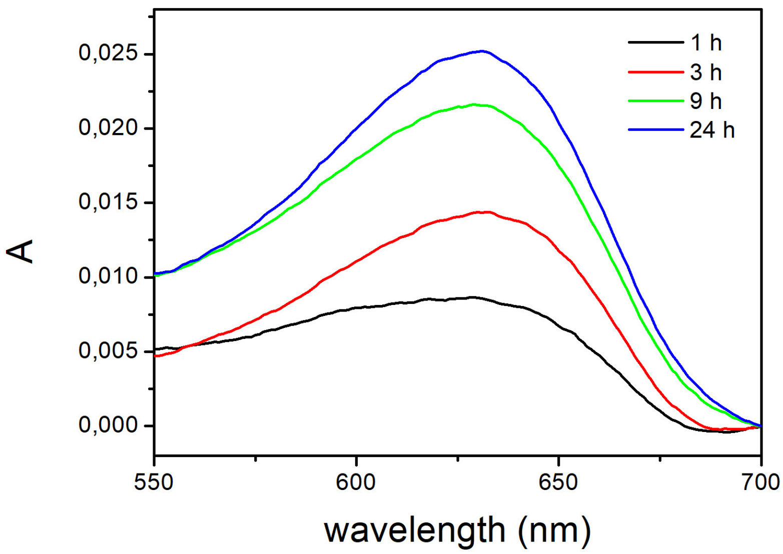

Figure S3. *A*bsorption spectra after reaction with anthrone of trehalose extracted from chromatophore samples incubated in 0.8 M trehalose for different times.

The absorption spectra obtained from the reaction between trehalose, extracted from chromatophore samples incubated for different times in 0.8 M trehalose, and anthrone are shown in Figure S3. The method is detailed in the protocol reported in section S2. In order to exclude trehalose possibly adsorbed nonspecifically on the outer membrane of the chromatophores, the spectra were corrected for the spectrum obtained from a chromatophore sample eluted in a Sephadex column immediately after mixing 1:1 (v/v) the chromatophores suspension (at a total bacteriochlorophyll concentration of 2 mM) with the 1.6 M trehalose solution.

From the corrected spectra, the absorbance value at 700 nm was subtracted from the value of the absorption at 624 nm. The $\Delta A_{624\text{-}700nm}$ values thus obtained were used to calculate the mass of trehalose using the calibration curve presented in section S2.

S4 Comparison of the effects of trehalose and sucrose on the carotenoid shift third phase.

Comparison between the effects exerted by sucrose and trehalose on the onset kinetics of carotenoid shift. Chromatophores were incubated in the presence of 0.8M sucrose or trehalose for three hours before starting the measurements.

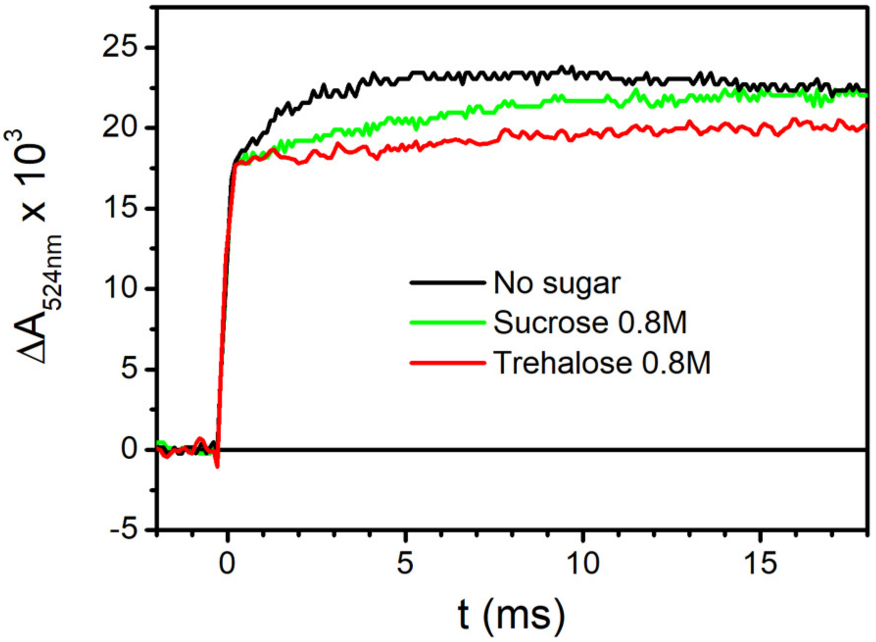

Figure S4. Kinetics of the carotenoid shift induced by a sinlge turnover flash on cromathopore pre-incubated for three hours in the presence of 0.8M sucrose (green trace) or in the presence of 0.8M trehalose (red trace). The control trace (black) has been acquired in chromatophores in the absence of sugar. Traces are the average of 2 signals. A minimum dark time of 60 s was allowed between repetitive photoexcitation during averaging. The other measuring conditions are described in section 4.3

In the presence of 0.8M sucrose (red trace), a slowdown in the raise of the carotenoid shift third phase (i.e. immediately after the steeper ΔA increase due to the first and second carotenoid shift phases) is observable as compared to the control. However the effect of sucrose (green trace) is significantly less severe than that observable in the presence of 0.8M trehalose.
